# NO/NOS system dysregulation as a key molecular mechanism in chemotherapy-induced cardiotoxicity: a review

**DOI:** 10.3389/fcvm.2025.1682471

**Published:** 2025-11-14

**Authors:** András Nagy, Zoltán Virág, Viktória Kiss, Denise Börzsei, Csaba Varga, Renáta Szabó

**Affiliations:** Department of Physiology, Anatomy, and Neuroscience, Faculty of Science and Informatics, University of Szeged, Szeged, Hungary

**Keywords:** cardiotoxicity, cardiovascular system, chemotherapy, nitric oxide synthase, NO signaling

## Abstract

Cardiotoxicity, as a side effect of chemotherapeutic treatments, represents a major limiting factor during anti-tumour therapies. This is usually evident by decreased cardiac function, and the cardiovascular diseases linked to chemotherapeutic agents can range from mild arrhythmias and hypertension to myocardial injuries and heart failure. One of the most important regulators of cardiovascular function is nitric oxide (NO), a small signalling molecule associated with multiple cardioprotective properties. The activity of NO producing enzymes, the nitric oxide synthases (NOSs), is tightly regulated by pathways responsible for maintaining the cardiovascular NO homeostasis. Thus, the dysregulation of the NO/NOS system could lead to severe pathologic changes in cardiomyocytes and endothelial cells. This narrative review examines the evidence on how chemotherapeutic agents affect cardiac NOS activity and NO signalling, and explores whether NO/NOS dysregulation may be considered as a relevant mechanism in the development of chemotherapy-induced cardiotoxicity.

## 1 Introduction

According to the World Health Organization, cancer and cardiovascular diseases (CVDs) both represent major health issues, accounting for nearly half of all deaths worldwide (1, 2). Although the options for cancer treatment are continuously evolving and improving the life expectancy of patients, chemotherapy-induced toxicities emerged as an unfortunate side effect of tumour treatment procedures. These include, but are not limited to: cardiotoxicity (CTX), central nervous system toxicities, peripheral neuropathy, nephrotoxicity, mucositis, electrolyte disorders, anaphylaxis and extravasation (3).

CTX in particular has been a major point of interest in this area, leading to the development of cardio-oncology. CTX is most often defined as a decrease of at least 10% in left ventricular ejection fraction (LVEF) to a value below 50%–55%; however, it is important to note that impaired cardiac function may be present before there are significant changes in LVEF, and also, the absence of LVEF decrease does not automatically exclude the presence of subclinical myocardial dysfunction (4, 5). CTX can be reversible (type II) or irreversible (type I), with clinical manifestations ranging from asymptomatic left ventricular dysfunction, hypertension and arrhythmias to myocardial ischaemia and congestive heart failure (CHF) (6). In one of our previous reviews, we discussed some of the more well researched, CTX-inducing chemotherapeutic agents, including: anthracyclines, a group of tetracyclic aglycone base containing cytotoxic antibiotics; antimetabolites, capable of interfering with DNA synthesis; alkylating agents, a subgroup of nitrogen mustards; microtubule inhibitors (MTIs), regulators of tubulin metabolism and tyrosine kinase inhibitors (TKIs), disruptors of cellular signalling pathways mediated by tyrosine kinase enzymes (7). There is a large variety of known molecular pathways and mechanisms of action which play a role in the development of chemotherapy- induced CTX, and these can often change depending on the biochemical properties of specific drugs. For example, anthracyclines, which stand on the forefront of cardio-oncology studies, are well known to pose a risk for developing type I CTX by generating reactive oxygen species (ROSs) through redox cycling and inducing inflammatory processes, which in turn cause mitochondrial dysfunction and DNA damage, leading to cardiomyocyte apoptosis and myocardial injury (8). On the other, trastuzumab, a monoclonal antibody (mAb) that targets the epidermal growth factor receptor-2 (HER2), inhibits normal neuregulin (NRG) function and dysregulates cellular survival signalling associated with HER2 (9). This reduces resistance against oxidative stress leading to DNA breaks and activation of mitochondrial apoptotic pathways but, unlike anthracyclines, trastuzumab is not directly responsible for the cellular damage, and usually causes reversible CTX (9).

One aspect that we believe might play a crucial role in the development of cardiac disorders related to chemotherapy, is the dysregulation of the nitric oxide synthase (NOS) system and the subsequent dysfunction in nitric oxide (NO) synthesis. Not only is NO an essential signalling molecule in the brain, that helps neurons to adapt to physiological changes (10), but it is also one of the most important mammalian vasodilators with a crucial role in protecting the cardiovascular system against the development of CVDs, by lowering blood pressure, regulating vascular tone, reducing platelet aggregation and leukocyte adhesion, and preventing the rapid reproduction of smooth muscle cells (11). In the last 15–20 years there has been a growing number of evidence suggesting that the interaction between CTX-inducing chemotherapeutic agents and NOSs leads to NO imbalance and a loss of its cardioprotective functions (12).

The main objective of this narrative review is to clarify the importance of NO and NOS function disruption caused by chemotherapeutic drugs in the development of CTX. Although some reviews do acknowledge the role of NO/NOS dysregulation as a mechanism involved in chemotherapy-induced CTX, it is either in regards to just one specific group of anticancer drugs (13, 14) or presented as one of many contributing factors to drug associated cardiac side effects, and not as a central element linking these factors together (15, 16). Our aim is to discuss the cardioprotective role of NO, and the mechanisms through which chemotherapeutics might alter this protection, leading to cardiac complications with the idea that NO/NOS dysregulation may act as a key molecular irregularity in most CTX inducing chemotherapeutic agents, bridging the gap between oxidative and nitrosative stress, loss of cardioprotective functions and apoptosis.

## 2 CTX-inducing chemotherapeutic agents

As we previously mentioned in our introduction, a large variety of chemotherapeutic agents are linked to the development of cardiovascular issues. There are some overlapping mechanisms associated with drug-induced CTX, such as the activation of proinflammatory processes, oxidative stress or the induction of apoptotic pathways (17, 18). Moreover, there is also a wide spectrum of more specific paths, through which different chemotherapeutics produce cytotoxicity, leading to side effects like CTX. Without being an exhaustive list, Table 1 presents the main mechanisms of action of chemotherapeutic agents often associated with the development of cardiac dysfunctions.

**Table 1:** Chemotherapeutic groups associated with CTX and their mechanism of action.

Chemotherapeutic groups	Notable examples	Main mechanisms of action	References
Anthracyclines	Daunorubicin, Doxorubicin, Epirubicin, Idarubicin, Pirarubicin	Inhibition of topoisomerase 2-β Double strand DNA breaks ROS generation through redox cycling of the quinone structure Disruption of the mitochondrial electron transfer chain, after binding to cardiolipin	(132–137)
Antimetabolites	Methotrexate, 5-FU, Capecitabine, Cytarabine	Cytostasis Prohibition of nucleotide precursor biosynthesis through the competitive inhibition of dihydrofolate reductase Prohibition of DNA synthesis via the inhibition of thymidylate synthase to transform dUMP into dTMP Prevention of DNA replication via competitive inhibition of DNA-polymerase Increased fluorocitrate level	(89, 138–144)
Alkylating agents	Cyclophosphamide, Ifosfamide, Cisplatin	DNA cross-linking Alkylation or covalent bonding to the N7 nitrogen atom of guanine Inhibition of DNA separation, replication and repair	(144–147)
MTIs	Paclitaxel, Docetaxel, Vinblastine, Vincristine, Vinorelbine, Vindesine, Vinflunine	Taxanes stabilize microtubule structures and inhibit their depolymerization Vinca alkaloids prohibit microtubule genesis through the inhibition of tubulin polymerization	(148–151)
TKIs (small molecule TKIs and monoclonal antibodies)	Imatinib, Lapatinib, Sunitinib, Sorafenib, Trastuzumab, Pertuzumab, Bevacizumab	Disruption of molecular pathways associated with cell proliferation and survival, through the dysregulation of VEGF, PDGF, EGF, VEGFR, PDGFR and HER-2 downstream signalling Downregulation of MAPK and PI3K/Akt pathways Induction of apoptosis through the inhibition of BCR-ABL tyrosine kinase Decreased mitochondrial potential	(152–155)

ROS, reactive oxygen species; 5-FU, 5-Fluorouracil; dUMP, deoxyuridine monophosphate; dTMP, deoxythymidine monophosphate; VEGF, vascular endothelial growth factor; PDGF, platelet-derived growth factor; EGF, epidermal growth factor; VEGFR, vascular endothelial growth factor receptor; PDGFR, platelet-derived growth factor receptor; HER-2, epidermal growth factor receptor-2; MAPK, Mitogen-activated protein kinase; PI3K/Akt, phosphatidylinositol 3-kinase/protein kinase B.

## 3 The NO/NOS system

### 3.1 NO synthesis and NOS structure

NO is a colourless gas and a free radical with an unpaired electron, that did not have any known biological relevance until the 1980s. This all changed in 1987, when both Ignarro et al. and Palmer et al. demonstrated that NO is in fact one of the most important mammalian vasodilators, previously known as the endothelium-derived relaxing factor (19, 20). Not long after this initial breakthrough the process of endogenous, enzymatic synthesis of NO from L-arginine (L-Arg) was also described (21), and the discovery of NO's role as a neuronal messenger led to the distinction between the three NOS isoforms: neuronal NOS (nNOS, NOS1), inducible NOS (iNOS, NOS2) and endothelial NOS (eNOS, NOS3) (22, 23).

These isoforms all share similar composition and way of function. NOS isoforms have a homodimeric structure, with each monomer possessing a C-terminal reductase- and an N-terminal oxygenase domain, with a calmodulin-binding region connecting them, and these domains are responsible for facilitating the electron transfer that leads to NO synthesis (24). Nicotinamide adenine dinucleotide phosphate (NADPH) serves as the primary reducing power, donating an electron that travels through both flavin adenine dinucleotide (FAD) and flavin mononucleotide (FMN) contained within the reductase domain before arriving to the heme group of the other monomer's oxygenase domain. Here, with the help of tetrahydrobiopterin (BH_4_), which binds to the heme group and is essential to homodimerization, L-Arg is oxidized in a two-step process that ends with the production of L-citrulline (L-Cit) and NO (25). Finally, diffusing through intracellular spaces, NO reaches and activates its most important effector, the soluble guanylyl cyclase (sGC); however, Kleschyov et al. present a plausible hypothesis regarding sGC activation, in which they argue that NO-ferroheme species (bound or not to a protein carrier) are a more likely NOS-derived signalling product than free NO itself (26, 27).

**Figure 1:**
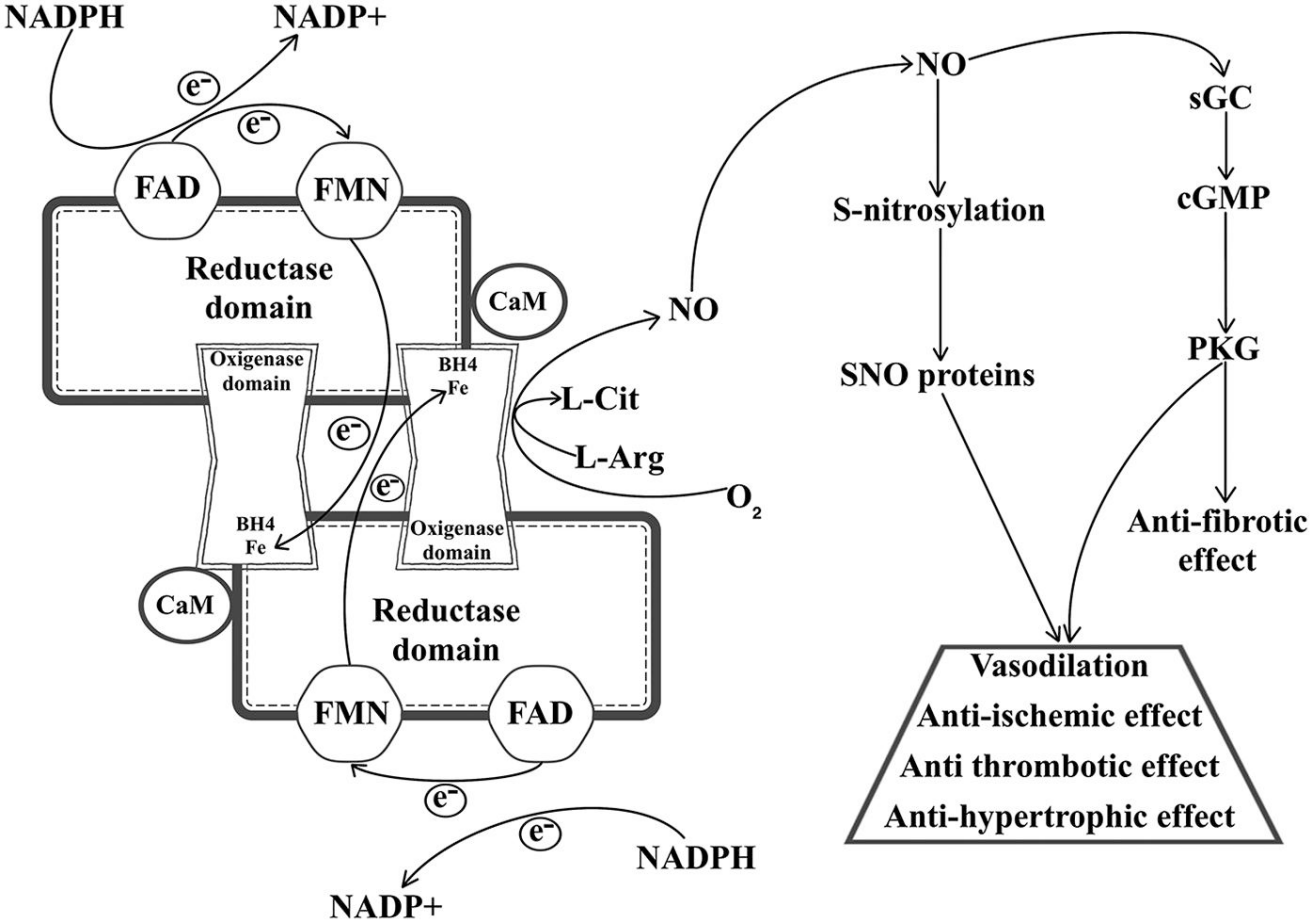
NO synthesis and the cardioprotective effects of NO signalling. NADPH, reduced nicotinamide adenine dinucleotide phosphate; NADP^+^, oxidized nicotinamide adenine dinucleotide phosphate; FAD, flavin adenine dinucleotide; FMN, flavin mononucleotide; CaM, calmodulin; BH4, tetrahydrobiopterin; L-Cit, L-citrulline; L-Arg, L-arginine; NO, nitric oxide; SNO, S-nitrosothiol; sGC, soluble guanylyl cyclase; cGMP, cyclic guanosine monophosphate; PKG, cGMP-dependent protein kinase type I; VSMC, vascular smooth muscle cell.

Looking beyond the basic structure and NO synthesizing function presented on Figure 1, the three isoforms show substantial differences as well, with respect to their subcellular localization and the physiological role of the NO produced by them. Canonically eNOS is mainly found in endothelial cells, where NO generation helps with smooth muscle relaxation and consequent vasodilation; nNOS is typically expressed close to synaptic clefts, where it can regulate the cyclic guanosine monophosphate (cGMP)-dependent release of neurotransmitters; and iNOS is mostly expressed in immune or epithelial cells, and responds to immunological stress (28). However, this does not mean that different NOSs cannot be present or modulate physiological processes in the same tissue or cells. The best example of this comes from cardiomyocytes, where the compartmentalization of eNOS and nNOS is responsible for opposing responses in NO mediated Ca^2+^ level regulation and contractility. During and after its translation, eNOS is myristoylated and palmitoylated (29), which enables the enzyme to anchor to caveolae, where it interacts with L-type Ca^2+^ channels, inhibiting Ca^2+^ uptake induced by β-adrenergic signalling. In contrast, nNOS, which is located close to the sarcoplasmic reticulum increases ryanodine receptor mediated intracellular Ca^2+^ release, thus enhancing cardiomyocyte contractility (30).

### 3.2 Protective mechanisms of NO in the cardiovascular system

NO has two major roles in the cardiovascular system: the regulation of the cGMP/cGMP-dependent protein kinase type I (PKG) signalling pathway and S-nitrosylation. NO is a direct activator of sGC, that turns guanosine triphosphate into cGMP, which phosphorylates PKG, and this kinase interacts with a myriad of targets related to cardiac function, including: cardiac myosin-binding protein C, cardiac troponin I (cTnI), titin, cysteine-rich LIM-only protein 4, vasodilator-stimulated phosphoprotein (VASP), cardiac L-type Ca^2+^ channel, cardiac ryanodine receptor, phospholamban, regulator of G-protein signalling 2/4, transient receptor potential canonical 6, GATA-binding protein, inhibitor of nuclear factor kappa-light-chain-enhancer of activated B cells (NF-κB inhibitor, IκBα), tuberin, ATP-sensitive K^+^ channel, Ca^2+^- and voltage activated K^+^ channel and proteasome regulatory particles and more (31). S-nitrosylation is a post translational modification which describes the process of covalent bonding between a cysteine (Cys) thiol of a protein and NO, forming an S-nitrosothiol (SNO). In a similar manner to cGMP/PKG signalling, this affects multiple cardiovascular function associated proteins, such as: β-arrestin 2, caspase-3 (CASP3), hypoxia-inducible factor 1-alpha (HIF-1α), mitogen activated protein kinase 7, N-ethylmaleimide-sensitive factor (NSF), ryanodine receptor 2, metallothionein, tissue transglutaminase (32). Thus, either through these effectors or the cGMP/PKG-pathway, NO is capable of mediating a considerable number of cardioprotective actions, as presented in Figure 1. One of the better characterized effects of NO is its capability to regulate vasodilation and blood pressure through the cGMP/PKG pathway. PKG is a pleiotropic enzyme and its activation through phosphorylation in vascular smooth muscle cells (VSMCs) leads to decreased intracellular Ca^2+^ concentration and reduced myosin light chain phosphorylation, resulting in decreased vascular tone and lower blood pressure (33). S-nitrosylation and S-denitrosylation were also shown to have a role in vascular tone regulation. Beigi et al. demonstrated that the lack of S-denitrosylation via S-nitrosoglutathione reductase (GSNOR) and high levels of S-nitrosoglutathione in GSNOR deficient mice resulted in systemic vasodilation, and blood pressure was kept in normal range through increased cardiac output (34). Another cardioprotective mechanism regulated by both PKG and S-nitrosylation is the inhibition of smooth muscle cell proliferation. On the one hand, PKG phosphorylates VASP at serine (Ser) 239, decreasing VSMC proliferation and injury related changes in phenotype (35). On the other, NO is directly responsible for the S-nitrosylation and subsequent inactivation of Ras homolog family member A, a small GTPase, which leads to antiproliferative extracellular signal-regulated kinase (ERK) signalling (36). Moreover, NO is essential in the regulatory process of platelet aggregation and displays a protective effect against the development of thrombosis. This is mainly attributed to PKG-dependent phosphorylation of small G-proteins, regulators of G-protein signalling and VASP, which inhibit intracellular Ca^2+^ release, integrin activation, cytoskeletal remodelling and granule secretion, preventing platelet activation and limiting thrombosis formation (37). Additionally, according to Morrell et al., the S-nitrosylation of NSF prohibits exocytosis of dense granules, lysosomal granules, and α-granules from human platelets, thus complementing PKG's antithrombotic role (38). In case of fibrosis, however S-nitrosylation of the heat shock protein 90 (HSP90) and the c-Jun N-terminal kinase (JNK) activates the transforming growth factor-β (TGF-β)/mothers against decapentaplegic homolog 3 (SMAD3) and JNK pathways, accelerating cardiac fibrosis (39, 40). Conversely, Qin et al. demonstrated that increasing eNOS activity and inducing NO/cGMP/PKG signalling inhibits cardiac fibrosis development (41). In addition, NO promotes antifibrotic and antihypertrophic effects by reducing endothelin-1 (ET-1), angiotensin II, and aldosterone activity in fibroblasts and myofibroblasts, and norepinephrine activity in cardiomyocytes, as well as by inactivating nuclear factor of activated T-cells signalling via PKG (42). Last, but not least, prevention and protection against ischaemia and ischaemia reperfusion injury through ischaemic pre- and postconditioning (IpreC and IpostC) is also highly mediated by NO. IpreC and IpostC are short and non-lethal episodes of cardiac ischaemia, induced either before or after an acute ischaemic event, with the explicit purpose to prevent or mitigate myocardial reperfusion injury (43). Both NOS-sGC signalling and S-nitrosylation are associated with IpreC and IpostC, mainly because of their capability to activate protein kinase C (PKC), activate mitochondrial K_ATP_ channel and inhibit mitochondrial permeability transition pore opening, resulting in mitochondrial preservation (44, 45). Interestingly, some evidence suggests that the inhibition of the NO/cGMP pathway reduces the cardioprotective effect of IpostC (46), meanwhile, in IpreC the selective inhibition of sGC or PKG does not limit cardioprotection, suggesting that IpreC might be mainly affected by S-nitrosylation and protein SNOs rather than the NOS signalling cascade (47).

### 3.3 Biochemical changes caused by the dysregulation of the NO/NOS system

Under normal circumstances NO synthesis is coupled to NADPH oxidation and the subsequent electron transport from the reductase domain to the oxygenase domain. This is facilitated by adequate concentrations of L-Arg, the primary substrate for the oxygenase domain, and BH_4_, which stabilizes the homodimeric structure of NOS, acts as an electron donor for O_2_ and catalyses L-Arg oxidation. NOS uncoupling refers to a state in which the electron transport induced by NADPH oxidation is separated from L-Arg oxidation, resulting in the production of the highly reactive superoxide (O_2_^−^) (48). eNOS uncoupling has been strongly associated with the development of endothelial dysfunction and CVDs, such as CHF, myocardial infarction and ischaemic cardiac damage (49). Oxidative stress, a phenomenon strongly associated with drug-induced CTX, plays a major role in eNOS uncoupling and dysregulation, inducing a vicious cycle which starts with increased intracellular ROS generation. O_2_^−^ is capable of quickly reacting with NO to form peroxynitrite (ONOO^−^), which causes nitrosative stress, that, together with oxidative stress inhibit BH_4_ regeneration by reducing dihydrofolate reductase (DHFR) activity, therefore depleting the cofactor's bioavailability and leading to even more O_2_^−^ production through uncoupled eNOS (50). It is important to mention, that overproduction of NO not only leads to increased levels of ROS and ONOO^−^, but also to the dysregulation of NO/cGMP signalling through a self-limiting feedback mechanism which inhibits sGC activity (51).

Although eNOS uncoupling and the subsequent decline in NO bioavailability due to oxidative stress are well established biochemical changes related to CVDs, the dysregulation of iNOS activity is also highly relevant (52). NO generated by iNOS acts as a downstream effector for interleukin-6 (IL-6) proinflammatory signalling, mediated by the Janus kinase (JAK)/signal transducers and activators of transcription 3 (STAT3) pathway. Yu et al. demonstrated that adult ventricular cardiomyocytes treated with IL-6 show significant increase in STAT3 phosphorylation at tyrosine 705 accompanied by increased iNOS synthesis and NO production, as well as significantly decreased contractility (53). Other findings also accentuate the role of NO overproduction facilitated by iNOS in myocardial infarction. Wilmes et al. found that cardiac tissue obtained by deceased individuals who were affected by acute infarction showed significantly increased iNOS expression, compared to healthy tissue samples, while also emphasizing the role of iNOS hyperactivity in the development of oxidative cardiac environment characterized by O_2_^−^ and ONOO^−^ production (54). Furthermore, according to Arvunescu et al. alongside with other proinflammatory mediators such as tumour necrosis factor-α (TNF-α), interleukin-1, IL-6, fibrinogen, C-reactive protein and myeloperoxidase (MPO), iNOS activation also leads to inhibition of cardiac contractility and induction of oxidative stress through NO-related cytotoxicity, playing a major role in the pathophysiology of heart failure with preserved ejection fraction and ischaemic cardiomyopathy (55).

Similarly to eNOS and iNOS activity, protein S-nitrosylation is also heavily susceptible to oxidative stress present in the cardiovascular system, and its dysregulation results in a loss of cardioprotective functions, leading to the development of CVDs such as heart failure (56). Moreover, S-nitrosylation of nicotinamide adenine dinucleotide (NADH) dehydrogenase 3 at Cys 39 and tripartite motif-containing protein at Cys 72 facilitate cardiac protection against ischaemic myocardial injury and post-infarct heart failure, while increased S-nitrosylation of muscle LIM protein at Cys 79 is associated with the pathogenesis of cardiac hypertrophy, through the activation of receptor-interacting protein kinase 3 and NOD-like receptor pyrin domain containing 3 (57). Additionally, eNOS mediated S-nitrosylation of the N-ethylmaleimide sensitive fusion protein (NSF) in vascular endothelial cells leads to reduced exocytosis of Weibel-Palade bodies, resulting in the inhibition of platelet and leukocyte attachment to post-capillary venule endothelium and a general anti-inflammatory effect (58). Furthermore, S-nitrosylation is a key regulator of NOS activity itself. Particularly, the enzyme activity of eNOS is limited by S-nitrosylation of Cys residues in the zinc-tetrathiolate cluster, which leads to destabilization of the dimeric structure and decrease in NO production (59, 60).

## 4 The impact of chemotherapeutic agents on the cardiovascular NO/NOS system

### 4.1 Anthracyclines

Anthracyclines are a group of cytotoxic antibiotics which enact their anti-tumour effect through multiple mechanisms, including DNA damage facilitated by topoisomerase-2β inhibition and dysregulation of the mitochondrial electron transfer chain. These drugs, and doxorubicin (DOX) specifically, can enter the cycle of NOS uncoupling and ROS generation at multiple points, resulting in CTX induced by oxidative and nitrosative stress. The quinone moiety in their structure can be reduced to semiquinone, which reduces molecular oxygen to regenerate back into quinone, creating O_2_^−^ in the process. This reaction is facilitated by multiple oxidoreductases, including: NADPH oxygenase (NOX), cytochrome P450 reductase, NADH dehydrogenase, xanthine dehydrogenase, and importantly eNOS itself (61, 62). Vasquez-Vivar et al. were the first ones to demonstrate that DOX is capable of directly binding to the reductase domain of eNOS, leading to uncoupled enzymatic activity, increased O_2_^−^ and decreased NO production (63). In addition, the proximity of NO and O_2_^−^ synthesis results in increased ONOO^−^ generation, further depleting NO and BH_4_ bioavailability (64, 65). Using NG-nitro-L-arginine methyl ester (L-NAME), a non-specific NOS inhibitor, Bahadır et al. found that decreasing NOS activity during DOX treatment significantly reduces DOX-induced CTX in 12-week-old male Sprague Dawley rats. A cumulative DOX dose of 15 mg/kg administered over 5 weeks (one injection per week) led to myocyte oedema, vacuolization in myocytes and myofibrillar loss in 50%, and interstitial oedema in 83% of the treated rats, as well as mitochondrial damage in 100% of animals. Moreover, DOX also significantly increased plasma malondialdehyde (MDA) concentration compared to controls, suggesting increased levels of lipid peroxidation. Conversely, 30 mg/kg L-NAME injection presented significant protection against these pathologic changes, emphasizing the risk posed by NOS malfunction during anthracycline treatment (66). However, other findings suggest, that selective inhibition of certain NOS isoforms yields better results regarding survival, compared to the non-specific mechanism of L-NAME. Following DOX treatment, with a cumulative dose of 12 mg/kg over 4 weeks, on different type of NOS knockout mice, Deng et al. observed that mortality in eNOS knockouts was only 10%, compared to 70% in nNOS knockouts, over a period of 60 days after DOX treatment (67). The importance of eNOS in the development of anthracycline induced CTX is substantiated by clinical findings as well. With the involvement of 176 women who had breast cancer and no prior CVDs of any kind before starting chemotherapy, Grakova et al. found that treatment involving DOX (either in combination with cyclophosphamide or with cyclophosphamide and docetaxel) led to the development of CTX in 52 patients, who showed significant decreases in LVEF, brachial artery diameter, endothelium-dependent and -independent vasodilatory capacity and significantly increased N-terminal prohormone of brain natriuretic peptide (NT-proBNP) and ET-1 levels after 12 months. Importantly, there was a significant association between the development of CTX and the presence of T/T genotypes for both the NOS3 (rs1799983) and the NADPH oxidase (rs4673) genes (68), further emphasizing the presence of ROS-initiated eNOS malfunction in anthracycline induced CTX.

Anthracyclines also inhibit sGC activity, and subsequently decrease downstream cGMP signalling. Vandenwijngaert et al. demonstrated that 20 mg/kg DOX reduces NO-stimulated sGC activity by 20% in C57BL/6J mice, due to the loss of the enzyme's prosthetic heme moiety, caused by oxidative damage. Furthermore, after 12 weeks of DOX treatment (2 mg/kg once a week), genetically modified mice with cardiomyocyte-specific reduction of sGC activity showed significantly increased left ventricular end-diastolic and systolic internal diameters (LVEDD, LVESD), as well as significantly decreased fractional shortening (FS) and ejection fraction (EF) compared to both DOX-treated and untreated wild type animals. This was accompanied by significant increase in oxidative and nitrosative stress biomarker levels, such as ROS, 3-nitrotyrosine and MDA (69). These observations were confirmed by Zhao et al. who presented similar results examining both H9c2 cardiomyoblasts and male Sprague-Dawley rats. They found that pretreatment of cardiomyoblasts with the sGC activator BAY60-2770 (4-(((4-carboxybutyl) (2-(5-fluoro-2-((4′-(trifluoromethyl) biphenyl-4-yl) methoxy) phenyl) ethyl) amino) methyl) benzoic acid) significantly increases cell viability and the phosphorylated-VASP/VASP ratio, while inhibiting p53 phosphorylation at Ser 15 and decreasing Bcl-2-like protein (Bax)/B-cell lymphoma 2 (Bcl-2) ratio compared to cells treated only with DOX, thus presenting significant antiapoptotic effects. Moreover, increased activation of sGC prevented significant increase in mitochondrial ROS and 3-nitrotyrosine generation and decrease of mitochondrial membrane potential induced by DOX, displaying protection against oxidative stress. The *in vivo* results were also in line with these findings. A cumulative DOX dose of 20 mg/kg (3.33 mg/kg three times a week, for two weeks) significantly decreased both LVEF and FS, while pretreatment with 5 mg/kg BAY60-2770 1 h before each DOX injection prevented these changes (70). Similar results were presented by Chen et al., assessing the effectiveness of another sGC agonist, vericiguat, in 8-week-old SPF male Sprague Dawley rats. On the one hand, a cumulative DOX dose of 12 mg/kg (1 mg/kg twice a week, for two weeks) caused significant increase in systolic and diastolic blood pressure, LVEDD and left ventricular mass index, as well as significant decrease in interventricular septal end-diastolic thickness, left ventricular posterior wall end-diastolic thickness, EF, FS and cardiomyocyte size. The treatment also led to significantly increased NT-proBNP, MDA levels and Bax/Bcl-2 ratio, as well as decreased nuclear factor erythroid 2-related factor 2 and superoxide dismutase (SOD) levels. Importantly, DOX also caused significant decrease in NO concentration which in turn resulted in impaired sGC activation and low concentrations of cGMP and PKG, despite no significant decrease in sGC protein levels. On the other hand, 1 mg/kg/day of vericiguat (administered for 8 weeks) successfully prevented these pathologic alterations with the exception of the decreased NO levels, sGC being its downstream effector (71).

iNOS activity is also heavily impacted by the effects of anthracycline therapy, which leads to an increased inflammatory state in cardiac cells. This phenomenon is primarily mediated by the activation of nuclear factor kappa-light-chain-enhancer of activated B cells (NF-κB) through the phosphorylation and inhibition of IκBα. Following this, NF-κB is responsible for the upregulation of iNOS expression, NO release and NT formation, as well as for increased expression of proinflammatory cytokines, such as interleukin-1β (IL-1β), IL-6, TNF-α or monocyte chemoattractant protein-1 and decreased expression of anti-inflammatory cytokines, like interleukin-10 (72–74). Moreover, these cytokines are in turn further responsible for upregulating iNOS activity and causing nitrosative and oxidative cardiac damage. This is indicated by elevated levels of cardiac injury biomarkers, such as creatine kinase-myocardial band (CK-MB), lactate dehydrogenase (LDH), cTnI, heart-type fatty acid binding protein, aspartate transaminase; as well as reduced cardiac function, evidenced by decreased EF and FS (75–77). In addition, iNOS-induced nitrosative stress is associated with cardiac tissue damage, including: cytoplasmic vacuolization, myofibrillar loss and disarrangement, inflammatory cell infiltration, oedema, congestion and myocyte necrosis (74, 76, 78). This tissue damage is also linked to proapoptotic signalling initiated by NO overproduction (79). This is supported by the findings of Bagchi et al., who demonstrated that DOX is responsible for direct and indirect activation of iNOS, the latter being the result of endoplasmic reticulum (ER) stress. According to their research, DOX induces the dissociation of the Bip-ATF6-eIF2 complex, leading to ER stress and subsequent iNOS activation. This, combined with the upregulating effect of NF-κB on iNOS activates toll-like receptor 2, resulting in cardiomyocyte apoptosis (80). On the other hand, NO shows significant anti-apoptotic effect by targeting CASP3 with S-nitrosylation. Maejima et al. demonstrated that increasing bioavailable NO concentration and upregulating S-nitrosylation leads to significant decrease in CASP3-induced cardiomyocyte apoptosis, following DOX treatment (81). Thus, the negative impact of anthracyclines on NO bioavailability might result in increased CASP3 activity and myocardial apoptosis.

Overall, anthracyclines pose a risk for developing CVDs by attenuating the cardioprotective effects of NO and enhancing nitrosative stress and proapoptotic signalling in the cardiovascular system. These phenomena are initiated primarily by eNOS uncoupling and iNOS hyperactivity, resulting in the depletion of bioavailable NO and the production of highly cytotoxic ONOO^−^.

### 4.2 Antimetabolites

Antimetabolites include chemotherapeutic agents that alter DNA synthesis, thus inhibiting the uncontrolled proliferation associated with cancer cells. Similar to anthracyclines, antimetabolites can also heavily impact NOS activity and consequently cardiovascular functions. Kanduri et al. proposed multiple mechanisms for CTX associated with fluoropyrimidines like 5-Fluorouracil (-FU) or its prodrug, capecitabine, including myocardial ischaemia mediated by endothelial dysfunction, causing decreased NO release, platelet aggregation, fibrin formation, followed by coronary vasospasm induced by vasoconstriction; as well as direct cardiomyocyte injury caused by fluorocitrate, a cardiotoxic metabolite of 5-FU, which increases ROS production and inhibits antioxidant protective functions (82), potentially leading to further dysfunction in eNOS activity and NO production. In addition, low levels of bioavailable NO can further worsen endothelial dysfunction through attenuated S-nitrosyaltion and a marked decrease of S-nitrosoglutathione (156). In 2020 Muhammad et al. presented evidence for crosstalk between proinflammatory and oxidative signalling pathways induced by 5-FU, which led to inflammatory cell infiltration, oedema, focal necrosis, blood vessel congestion and sarcoplasm vacuolation in cardiomyocytes. According to their research, a weekly injection of 50 mg/kg 5-FU (administered for 6 weeks) enhanced both Rho-associated kinase (ROCK) activity and the ET-1/ERK pathways in male Wistar rats. This phenomenon has multiple negative effects on cardiac NO homeostasis, including the direct inhibition of eNOS by ET-1, as well as indirect inhibition mediated by ROCK, which prevents protein kinase B (Akt)-induced eNOS phosphorylation and activates NF-κB. Moreover, 5-FU increases NOX-derived ROS generation which also enhances ET-1 and ROCK signalling, worsening the oxidative imbalance and inflammatory state of cardiac cells, further decreasing eNOS derived NO levels (83). In addition to this, Refaie et al. showed that toll-like receptor signalling is also plays a role in antimetabolite-induced eNOS dysfunction. A single dose of 150 mg/kg 5-FU led to significant increase in cTnI, CK-MB and LDH levels, as well as sarcoplasm vacuolation, cardiac muscle fibre separation and degeneration, and inflammatory cell infiltration. These signs of cardiac injury were accompanied by upregulated toll-like receptor 4 (TLR4)/Myeloid Differentiation Primary Response 88 (MyD88) signalling, which not only resulted in decreased expression of eNOS and reduced NO levels, but also decreased the cellular total antioxidant capability (TAC) and activated both NF-κB and CASP3 function (84). The negative impact of 5-FU on the cardiac antioxidant system was also confirmed by Salomao et al., who observed significantly increased MPO activity and lipid hydroperoxide concentrations, as well as decreased SOD and catalase (CAT) activity, glutathione (GSH) and nitrate levels (85). On the other hand, DHFR inhibitor antimetabolites, such as methotrexate are responsible for eNOS dysfunction by limiting BH_4_ bioavailability in cardiomyocytes and endothelial cells. Both Ren et al. and Crabtree et al. demonstrated that methotrexate treatment inhibits DHFR's ability to convert dihydrobiopterin (BH_2_) into BH_4_, thus leading to eNOS uncoupling, which results in increased O_2_^−^ generation and decreased NO production (86, 87). However, we should also mention that some evidence suggests methotrexate having positive cardiovascular effects, specifically through the activation of the adenosine monophosphate (AMP)-activated protein kinase (AMPK)/eNOS pathway, thus reducing endothelial dysfunction (88).

Besides uncoupling eNOS, antimetabolites are also responsible for increasing iNOS derived NO production in cardiomyocytes, which is linked to nitrosative stress and inflammation. Gui et al., as well as Abukhalil et al. found that one dose of 150 mg/kg 5-FU leads to cardiac damage characterized by elevated CK-MB, cTnI and LDH levels, and also necrotic degradation, inflammation and vacuolation of myocardial tissue. These were accompanied by significantly decreased antioxidant parameters (SOD, CAT, glutathione peroxidase, GSH), and significantly increased levels of proinflammatory cytokines and transcription factors (IL-1β, IL-6, TNF-α, NF-κB), which in turn increased iNOS expression and resulted in NO overproduction, as well as proapoptotic signalling (89, 90). Similar mechanisms were found by Al-Taher et al. and Mahmoud et al. respectively, using one dose of 20 mg/kg methotrexate on male rats. The methotrexate treatment led to necrotic damage, fragmentation and vacuolation of the cardiac muscle, linked to significant increase in both oxidative stress and inflammation, and the subsequent increase in iNOS derived NO and TLR4/CASP3 mediated proapoptotic signalling (91, 92).

On the whole, by inhibiting DHFR activity, inducing oxidative imbalance and initiating proapoptotic signalling antimetabolites possess the capability to dysregulate the NO/NOS system and cause cardiac injuries.

### 4.3 Alkylating agents

Cyclophosphamide (CPA) and ifosfamide are nitrogen mustards that require bioactivation catalysed by P450 enzymes in order to form metabolites with DNA-alkylating capability. However, this process also results in the generation of acrolein, a secondary metabolite associated with a wide range of tissue toxicities (93). Thus, the use of these chemotherapeutic agents may affect cardiovascular health both directly and indirectly, through mechanisms mediated by acrolein.

One of these mechanisms appears to be the unsaturated aldehyde's inhibitory effect on PKC-ɛ activity. Wang et al. demonstrated that a single dose of 5 mg/kg acrolein disrupts NO mediated cardioprotective PKC-ɛ signalling in mice by decreasing the enzyme's mitochondrial expression and increasing the formation of acrolein-PKC-ɛ adducts, resulting in mitochondrial dysfunction. The subsequent CTX was characterized by significantly increased infarct size after coronary occlusion, compared to animals that did not receive acrolein treatment (94). In addition, Ismahil et al. investigated the effects of acrolein on cardiac function, as well as the differences between the acute and chronic effects of acrolein on eNOS function. They determined that 1 mg/kg acrolein, administered for 48 days leads to significantly increased LVEDD, LVESD and decreased velocity of circumferential fibre shortening and FS. Moreover, long term acrolein treatment resulted in eNOS uncoupling marked by significantly elevated concentration of NT and decreased levels of eNOS dimers, while a single dose of 5 mg/kg did not affect eNOS dimerization but significantly decreased its activity by inhibiting phosphorylation at Ser 1177 (95).

Additionally, just like anthracyclines and antimetabolites, alkylating agents take part in the development of cardiac injuries through increased iNOS-derived NO production. Using 200 mg/kg CPA on male Wistar rats, El-Agamy et al. observed elevated levels of cardiac injury serum biomarkers (CK-MB, LDH, cTnI and cardiac troponin T (cTnT)), as well as histopathologic changes, including: cardiac tissue degeneration, loss of muscular striation, vascular congestion, haemorrhage, infiltration of inflammatory cells and necrosis. These were accompanied by significantly increased cardiac NO concentration, attenuated SOD activity, decreased GSH concentration and increased MDA levels, implying elevated oxidative stress. Furthermore, cardiac inflammation and apoptosis was characterized by increased TLR4 signalling, as well as elevated concentrations of NF-κB, TNF-α, CASP3 and increased Bax/Bcl-2 ratio (96). These findings were later confirmed and further detailed by Iqubal et al., who found that one-time treatment of Swiss albino mice with 200 mg/kg CPA elevated CK-MB, LDH, cTnT and brain natriuretic peptide serum concentration and led to cardiac vacuolation, myofibrillar degeneration, pyknosis and fibrosis. The observed pathologic alterations were linked to oxidative and nitrosative stress, cardiac inflammation and apoptosis, which was associated with significantly increased NF-κB, TNF-α, IL-1β, IL-6 concentrations, and CASP3 signalling, both linked to significantly increased cardiac expression and activity of iNOS (97). Saleh et al. also presented similar results regarding the effects of iNOS activity in CPA-induced CTX. According to their research, one dose of 200 mg/kg CPA administered to male Wistar rats leads to cardiac injury, confirmed by significantly elevated serum CK-MB and LDH levels as well as congestion of myocardial blood vessels, myocyte necrosis, intramyocardial oedema and focal mononuclear cell infiltration. These changes were linked to significant decrease in regulatory T cell expression, which normally play an anti-inflammatory role, as well as significant increase in the cardiac expression of iNOS (98).

In essence, alkylating agents might cause adverse effects in the cardiovascular system, either by directly enhancing cardiac inflammation and programmed cell death, both linked to iNOS derived NO production; or indirectly through acrolein which reduces NO bioavailability, impairs PKC-ɛ mediated cardioprotection and causes mitochondrial dysfunction.

### 4.4 Microtubule inhibitors (MTIs)

MTIs are a group of chemotherapeutic agents which comprises drugs that either inhibit tubulin polymerization into microtubules or stabilize microtubules and prohibit their depolymerization (99). Although their effects on the NO/NOS system are not yet fully elucidated, there is some evidence to suggest that their mechanism of action might lead to CTX through the dysregulation of NOS activity. For example, according to Fitzpatrick and Wheeler, paclitaxel and docetaxel are responsible for the induction of proinflammatory gene transcription, leading to increased activity of TNF-α, IL-1β, IL-8 and elevated NO synthesis (100). Despite some findings showing that chronic low dose paclitaxel treatment can improve cardiac function in certain pathologies characterized by cytoskeletal alterations affecting cardiomyocytes (101), Malekinejad et al. found that that a weekly dose of 7.5 mg/kg paclitaxel (administered for 4 weeks) led to diffused oedema, haemorrhage, congestion and necrosis in the cardiac tissue, as well as significantly elevated serum CK-MB level and reduced TAC. These parameters were associated with increased lipid peroxidation and cardiac NO concentration (102).

However, other research suggests that the primary mechanism through which MTIs are capable of disrupting the integrity of the NO/NOS system is the inhibition and downstream dysregulation of the VEGF signalling pathway. Using human umbilical vein endothelial cells (HUVECs), Murtagh et al. demonstrated that docetaxel causes HSP90 ubiquitination and proteasomal degradation. This is particularly damaging in regards to VEGF signalling, not only because HSP90 stabilizes HIF-1α, which activates VEGF during hypoxia, but also because Akt and eNOS are HSP90 client proteins, dependent on the chaperon to maintain functional and structural integrity. Moreover, docetaxel also inhibited the phosphorylation of focal adhesion kinase (FAK) and the activation of integrin α_V_β_3_, two molecules that together with vascular endothelial growth factor receptor-2 (VEGFR-2) form a complex responsible for VEGF signal transduction by Akt phosphorylation, leading to significant reduction in eNOS phosphorylation and bioavailable NO production, thus resulting in endothelial dysfunction (103). Moreover, tubulin binding drugs, such as docetaxel or vincristine can also directly inhibit HIF-1α, limiting its capability as a VEGF activator (104). In addition, Ota et al. found that the expression of sirtuin 1 (Sirt1), a cell cycle regulator implicated in inducing HIF-1α nuclear translocation and Akt/eNOS phosphorylation (105, 106), is downregulated by paclitaxel. This led to significantly altered eNOS expression and the development of endothelial cell senescence (107).

In general, these findings attest that the disruptive capability of MTIs in regards to VEGF signalling, is associated with CTX mediated by inhibited eNOS function and NO production, as well as subsequent endothelial dysfunction.

### 4.5 Tyrosine kinase inhibitors (TKIs)

The tumour-suppressive effect of small molecule tyrosine kinase inhibitors (smTKIs), as their name suggest, comes from their capability to dysregulate the activity of several tyrosine kinases by blocking their ATP-binding site. Tyrosine kinases targeted by smTKIs include: VEGFR 1/2, platelet-derived growth factor receptor (PDGFR), and members of the human epidermal growth factor receptor family (epidermal growth factor receptor (EGFR or ErbB1), HER2, HER3, HER4) (108). mAbs are technically not TKIs since they target antibodies on the extracellular surface of tumour cell membranes (109). However, mAbs often associated with CTX, such as trastuzumab or bevacizumab, exert their effect through blocking the activation of receptor tyrosine kinases by preventing their dimerization or the binding of an extracellular ligand (e.g., HER2/HER4, VEGF/VEGFR) (110). Thus, for the purpose of this review, we decided to categorize them together with smTKIs.

The VEGF-induced biochemical cascade, mediated by VEGFR-2 signal transduction, represents a crucial signalling pathway in the preservation of cardiovascular health. VEGFR-2 regulates several crucial cellular phenomena, such as cell survival, cell migration, vascular cell permeability and enhancement of cell proliferation. It is also responsible for the induction of vasodilation via the upregulation of the phosphatidylinositol 3-kinase (PI3K)/Akt/eNOS pathway and the subsequent increase in NO production (111). Interestingly, imatinib, a drug that decreases PI3K/Akt/eNOS activity in a VEGF-independent manner, paradoxically shows some cardioprotective aspects, through PDGFR inhibition (112). Unfortunately, the use of chemotherapeutic agents that target VEGF signalling, like sunitinib, sorafenib or bevacizumab, may lead to cardiovascular complications (113–115). Hypertension specifically has been demonstrated multiple times to be one of these complications. In a phase 3 clinical trial Escudier et al. found that treatment with 400 mg/kg sorafenib twice a day, for a median duration of 23 weeks lead to a significant increase in the incidence of hypertension, affecting 76 out of 451 (16.85%) patients, with 44 (9.75%) people experiencing mild or moderate and another 16 (3.54%) experiencing severe or life threatening hypertension (116). Similarly, in another phase 3 trial Motzer et al. observed significantly increased rates of hypertension induced by 50 mg/day sunitinib, administered in 6-week cycles (4 weeks of treatment followed by 2 weeks without treatment) for a median duration of 6 months. 90 out of 375 (24%) people were affected by hypertension, with 30 (8%) showing signs of severe cases (117). In addition, evidence presented by Chu et al. shows that high incidences (47%) of sunitinib-induced hypertension (>150/100 mm Hg) correlate with an increased number of patients (20%) presenting LVEF lower than 50% and people (18%) with increased serum cTnI levels (>0.10 ng/mL). These changes were also associated with fatal (1%) and non-fatal cardiovascular events [myocardial infarction (1%), heart failure (8%)] (118). Inhibition of VEGF signalling by sunitinib has also been shown to alter eNOS function via Akt-independent mechanism as well. Using HUVECs, Shashar et al. demonstrated that VEGF activates the cationic amino acid transporter-1 (CAT-1) through VEGFR-2 signal transduction. CAT-1 is responsible to deliver L-Arg to eNOS, which can than increase NO production. Adding 50 or 100 ng/mL VEGF to the cell culture significantly increased CAT-1 concentration and activity, as well as nitrite/nitrate levels. However, 2 µM sunitinib negated this effect, proving that the VEGF inhibitor can alter eNOS derived NO production by dysregulating VEGFR-2/CAT-1 activity (119). The cardiotoxic effects of other VEGF inhibitors, like bevacizumab, are a little more ambiguous. On the one hand, Robinsons et al. reported significantly decreased cGMP and NO levels in patients using small molecule VEGF inhibitors, while bevacizumab resulted in no such decrease (120). On the other hand, according to Economopoulou et al., the inhibitory effect of VEGF signalling by bevacizumab is associated with increased incidence of hypertension (4%–35%), CHF (2%–4%) and venous thromboembolic events (3%–19.4%) (121). This is substantiated by a meta-analysis conducted by Zhu et al., who found that treatment with bevacizumab at low doses (3, 5 or 7.5 mg/kg/dose) induced hypertension, with an incidence ranging between 2.7% and 32%, while high doses (10 or 15 mg/kg/dose) were associated with an incidence between 17.6% and 36%. Moreover, severe hypertension appeared in 8.7% of patients who got low dose bevacizumab treatment, and in 16% of patients treated with high doses (122).

Beside VEGF, epidermal growth factor (EGF) and NRG signalling mediated by members of the HER family also play a major role in regulating NO production and maintaining normal cardiovascular function. Binding of EGFs and NRGs to their receptors induces HER heterodimerization, which is necessary for the activation of biochemical pathways that lead to cell proliferation, differentiation, survival and repair (123). NRGs specifically have a high affinity for binding to HER4, which initiates HER4/HER2 heterodimerization, and results in upregulation of Akt activity, which not only stimulates the function of anti-apoptotic pathways and reduces ROS generation, but also upregulates eNOS derived NO production (124, 125). Trastuzumab, the first monoclonal antibody specifically designed to inhibit HER2, prevents HER2/HER4 heterodimerization, thus prohibiting downstream NRG signalling. In cardiomyocytes this leads to downregulation of cellular survival pathways, increased oxidative stress through elevated NOX activity and reduced NO production, resulting in CTX and myocardial apoptosis (125). Although CTX induced by HER2 inhibition is not as severe as the effects of more aggressive chemotherapeutic agents, such as anthracyclines, and their negative effects on the cardiovascular system are usually reversible with the termination of treatment, they can, nonetheless, synergistically worsen the effects of other chemotherapeutics, mainly by the downregulation of eNOS activity and anti-apoptotic signalling. A prime example of this is the effect of double treatment with trastuzumab and DOX on cardiomyocytes. Using wild type and eNOS knockout mice, Zeglinski et al. demonstrated that a single dose of 10 mg/kg trastuzumab did not significantly alter parameters of cardiac function, such as LVEDD, LVEF% or endocardial velocity, neither has it induced histological changes. Contrary to this, a single dose of 20 mg/kg DOX or one 10 mg/kg dose of trastuzumab combined with 20 mg/kg DOX resulted in myofibrillar degradation and vacuolization, as well as significantly increased LVEDD and decreased LVEF% and endocardial velocity, with the combined treatment having a significantly stronger impact on every parameter, compared to DOX monotreatment. It was also observed that survival rates and oxidative stress were significantly worsened by eNOS knockout (126). Similar to mAbs, smTKIs targeting HER2 can also enhance the effects of anthracyclines. Hsu et al. demonstrated on a human pluripotent stem cell-derived cardiomyocyte model that a non-apoptotic concentration of lapatinib combined with DOX induces apoptosis by significantly upregulating iNOS activity and cytotoxic NO production (127).

Overall, smTKIs and mAbs may negatively impact cardiovascular health by prohibiting downstream signalling of tyrosine kinases which are involved with the activation eNOS and NO production or with pathways responsible for cardiomyocyte and endothelial cell protection.

## 5 Conclusions

**Figure 2:**
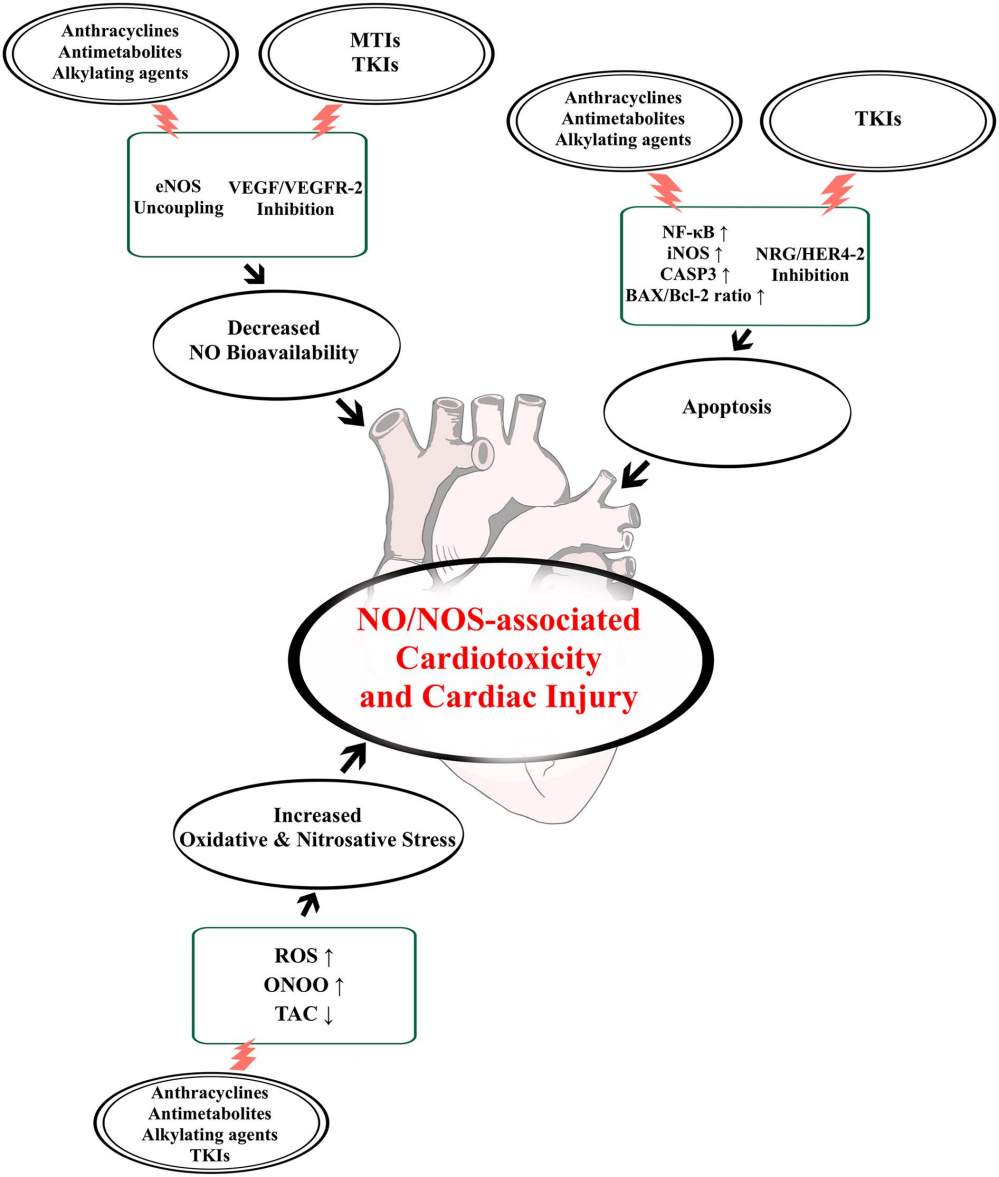
Chemotherapy-induced NO/NOS dysregulatory effects leading to cardiotoxicity and cardiac injury. Chemotherapeutic agent groups often associated with the development of cardiotoxicity and cardiac injury include: anthracyclines, antimetabolites, alkylating agents, microtubule inhibitors (MTIs) and tyrosine kinase inhibitors (TKIs). Their effects on the nitric oxide (NO)/nitric oxide synthase (NOS) system can lead to a loss of NO bioavailability through endothelial NOS (eNOS) uncoupling or inhibition of vascular endothelial growth factor (VEGF)/VEGF receptor-2 (VEGFR-2) signalling. Moreover, these drugs can also induce cardiovascular cell death, either by blocking neuregulin (NRG) and epidermal growth factor receptor-4 and -2 (HER4-2) function or by enhancing inducible NOS (iNOS) activity through nuclear factor kappa-light-chain-enhancer of activated B cells (NF-κB), leading to apoptosis mediated by caspase-3 (CASP3) and elevated Bcl-2-like protein (Bax)/B-cell lymphoma 2 (Bcl-2) ratio. Finally, these effects also lead to oxidative and nitrosative stress via increased reactive oxygen species (ROS) and peroxynitrite (ONOO^−^) generation, and decreased total antioxidant capability (TAC), thus damaging the heart even further.

To summarize, the NO/NOS system is a key regulator of cardiovascular function and is responsible for maintaining NO homeostasis in cardiomyocytes and endothelial cells. This, in turn, provides cardioprotective functions via NO bioactivity. The use of chemotherapeutic treatments, as seen on Figure 2, might play a role in the dysregulation of the NO/NOS system, and consequently result in the development of CTX mediated by NO imbalance. Our narrative review highlighted the potential role of such impairment as a key mechanism underlying cardiovascular dysfunction and deterioration associated with cardiotoxic chemotherapeutic treatments, both during and after therapy.

According to the currently available evidence, the functions of the NO/NOS system can be compromised in a few ways by the effects of anti-tumour therapies. These are mainly connected to the alteration of eNOS function, due to uncoupling or downregulated activity, leading to significant decrease in bioavailable NO, as well as to the upregulation of iNOS activity, which results in elevated NO production associated with cytotoxicity via apoptotic and proinflammatory signalling. These changes seem to interact with each other at the level of nitrosative stress mediated by ONOO^−^, a type of reactive nitrogen species formed by the reaction between O_2_^−^ and NO, and directly involved with cardiomyocyte and endothelial damage. Moreover, oxidative stress and mitochondrial dysfunction, as well as the inhibition of molecular cascades responsible for cellular survival, such as the VEGF or NRG pathways, further exacerbate the negative impact caused by the loss of NO regulated cardioprotective functions, finally resulting in cardiac injury and CTX.

One important limitation of our review comes from an apparent lack of clinical studies, which could bridge the gap between the data accumulated from animal research and application in human treatment. Despite current results from animal studies highlight NO signalling malfunctions induced by chemotherapeutic agents as plausible key dysregulators of cardiovascular health, differences in physiology, doses, and treatment lengths pose a complex obstacle for their implementation in clinical settings. That being said, there are also a handful of clinical studies and trials that analysed the mechanistic relevance of NO/NOS dysregulation in cancer therapy-associated CTX, and assessed its potential in the uncovering of predictive biomarkers and development of therapeutic options for chemotherapy related cardiac dysfunction. Early changes in arginine, asymmetric dimethylarginine and N-monomethylarginine concentrations have been shown to significantly correlate with rates of left ventricular systolic dysfunction in breast cancer patients receiving combined DOX and trastuzumab therapy (128). However, the use of well-established NO modulators in a cardio-oncologic perspective proves to be quite challenging. Sildenafil, a cGMP-specific phosphodiesterase type 5 inhibitor, showed no beneficial effects in preventing anthracycline-induced CTX during a phase I/II clinical trial, despite it being approved for the treatment of erectile dysfunction and pulmonary hypertension, due to its vasodilatory effect mediated by cGMP/PKG upregulation (129). Although, other approches related to the stabilization of NO homeostasis, such as BH4 or nitrate supplementation, resulted in positivie outcomes for patients with high blood pressure (130, 131), there is yet a lack of clinical trials assesing their efficacy in regards to cardiovascular symptoms caused by cancer treatments.

Taken together, these findings highlight the need for further research targeting the NO/NOS system as a potential therapeutic target for the prevention of chemotherapy-induced CTX.
